# Supplementary material for: Disrupting actin filaments enhances glucose-stimulated insulin secretion independent of the cortical actin cytoskeleton
Source: J Biol Chem. 2023 Oct 10;299(11):105334. doi: 10.1016/j.jbc.2023.105334 (PMC10641669; doi:10.1016/j.jbc.2023.105334)
Supplement: Supporting Figures S1–S3 [file mmc1.pptx]

## Slide 1
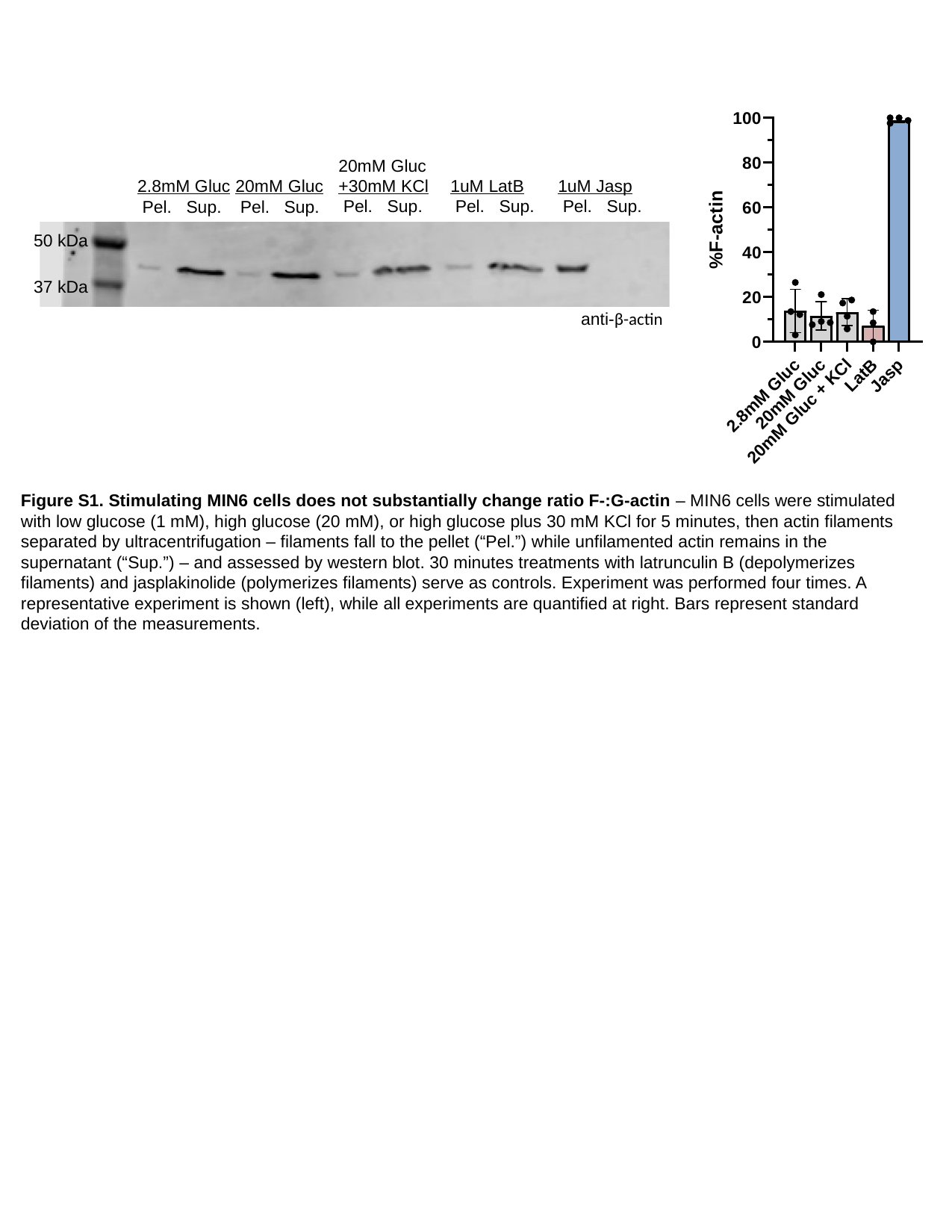

20mM Gluc
+30mM KCl
 Pel. Sup.
1uM LatB
 Pel. Sup.
1uM Jasp
 Pel. Sup.
2.8mM Gluc
 Pel. Sup.
20mM Gluc
 Pel. Sup.
50 kDa
37 kDa
anti-β-actin
Figure S1. Stimulating MIN6 cells does not substantially change ratio F-:G-actin – MIN6 cells were stimulated with low glucose (1 mM), high glucose (20 mM), or high glucose plus 30 mM KCl for 5 minutes, then actin filaments separated by ultracentrifugation – filaments fall to the pellet (“Pel.”) while unfilamented actin remains in the supernatant (“Sup.”) – and assessed by western blot. 30 minutes treatments with latrunculin B (depolymerizes filaments) and jasplakinolide (polymerizes filaments) serve as controls. Experiment was performed four times. A representative experiment is shown (left), while all experiments are quantified at right. Bars represent standard deviation of the measurements.

## Slide 2
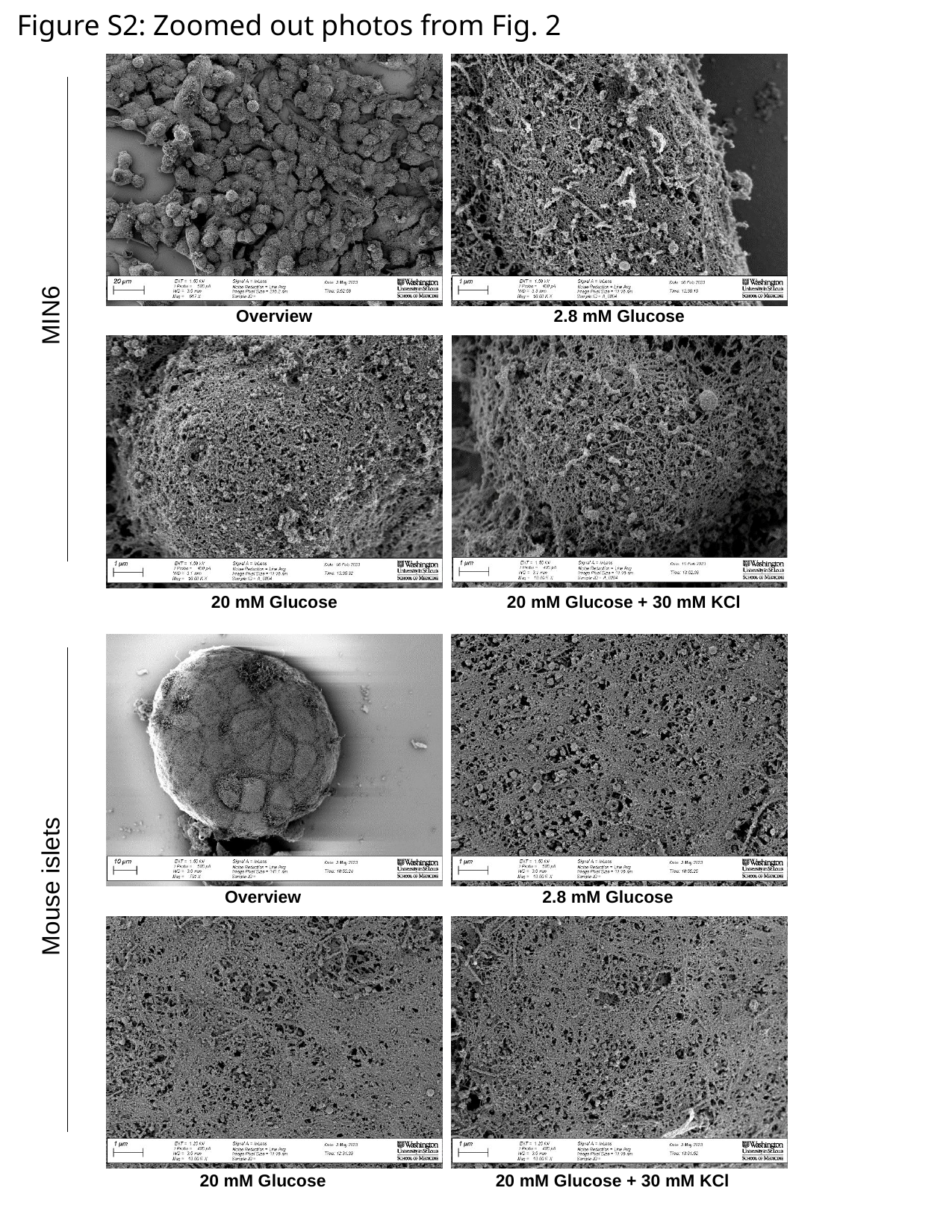

Figure S2: Zoomed out photos from Fig. 2
Overview
2.8 mM Glucose
20 mM Glucose
20 mM Glucose + 30 mM KCl
MIN6
Overview
2.8 mM Glucose
20 mM Glucose
20 mM Glucose + 30 mM KCl
Mouse islets

## Slide 3
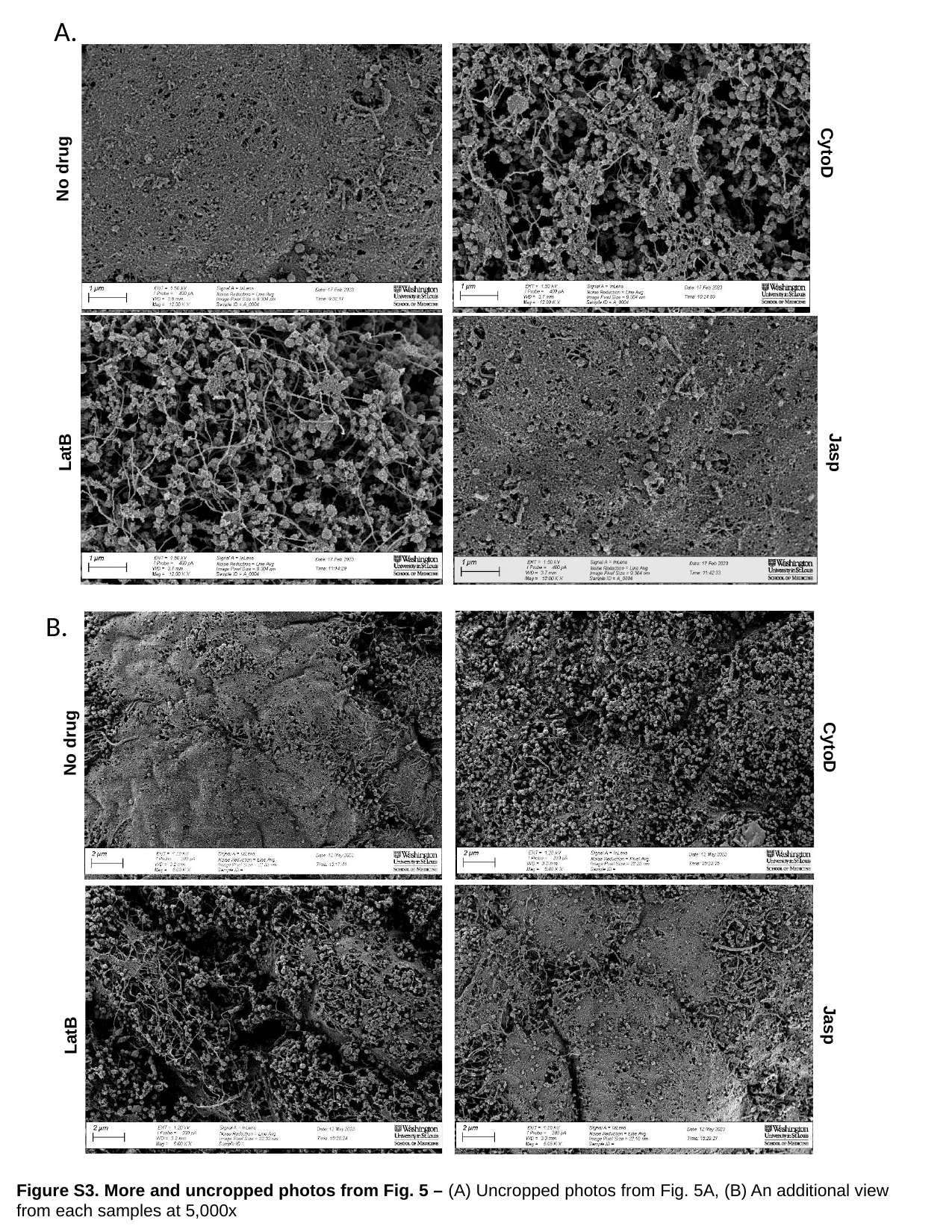

A.
CytoD
No drug
LatB
Jasp
B.
No drug
CytoD
Jasp
LatB
Figure S3. More and uncropped photos from Fig. 5 – (A) Uncropped photos from Fig. 5A, (B) An additional view from each samples at 5,000x
